# Male-derived transcripts isolated from the mated female reproductive tract in *Drosophila melanogaster*

**DOI:** 10.1093/g3journal/jkad202

**Published:** 2023-09-19

**Authors:** Julie M Cridland, David J Begun

**Affiliations:** Department of Evolution and Ecology, University of California, Davis, Davis, CA 95616, USA; Department of Evolution and Ecology, University of California, Davis, Davis, CA 95616, USA

**Keywords:** parovaria, spermatheca, seminal receptacle, expression

## Abstract

In species with internal fertilization, sperm, and seminal fluid are transferred from male to female during mating. While both sperm and seminal fluid contain various types of molecules, including RNA, the role of most of these molecules in the coordination of fertilization or in other possible functions is poorly understood. In *Drosophila*, exosomes from the accessory gland, which produces seminal fluid, are transferred to females, but their potential cargoes have not been described. Moreover, while the RNA composition of sperm has been described in several mammalian species, little work on this problem has occurred in *Drosophila*. Here we use single nucleotide polymorphism differences between males and females from a set of highly inbred lines of *D. melanogaster*, and transcriptome data from the female reproductive tract, sperm, testis, and accessory gland, to investigate the potential origin, male vs female, RNA molecules isolated from 3 female reproductive tract organs, the seminal receptacle and spermatheca, which store sperm, and the parovaria, which does not. We find that mated females carry male-derived transcripts from many genes, including those that are markers of the accessory gland and known seminal fluid proteins. Our observations also support the idea that intact sperm transcripts can be isolated from the female sperm storage organs.

## Introduction

Sexual reproduction is a collaborative enterprise, though conflicts between sexes may also arise (Parker and Others 1979). In animals with internal fertilization, including insects, males transfer sperm and seminal fluid during mating, both of which are necessary for successful reproduction. The main model system for investigating insect reproduction is *Drosophila melanogaster*. In this species, seminal fluid is necessary for proper storage of sperm in the female sperm storage organs and their subsequent use (reviewed in Avila *et al*. 2011), and also facilitates initiation of a suite of physiological and behavioral changes in females, known as the post-mating response, including stimulation of oviposition and suppression of remating behavior (Fowler 1973). A major component of *Drosophila* seminal fluid is the various proteins secreted by the paired accessory glands, the ejaculatory duct, and the ejaculatory bulb (reviewed in Wigby *et al*. 2020). Many of these proteins belong to a few biochemical classes, not only in *Drosophila* but in other species as well, including mammals (Wigby *et al*. 2020). Functional investigation of individual seminal fluid proteins (SFPs) in *D. melanogaster* has been carried out through the phenotypic analysis of knockout and RNAi knockdown animals (Herndon and Wolfner 1995; Ravi Ram *et al*. 2006; Ravi Ram and Wolfner 2007a, 2007b; Wong *et al*. 2008; Patlar and Civetta 2022). In addition to its role in promoting fertilization, seminal fluid may influence competitive interactions between the sperm of multiple males in the female reproductive tract (Harshman and Prout 1994; Clark *et al*. 1995; Fiumera *et al*. 2007; Wigby *et al*. 2020). Functionally significant polymorphism of genes affecting seminal fluid function is evident in the rapid evolution of male toxicity toward females and the rapid evolution of male sperm displacement phenotypes in experimental populations of *D. melanogaster* in which the mating system has been manipulated (Rice 1996; Holland and Rice 1999). The outcome of sperm competition is also influenced by female-expressed genetic variation and the interaction of genetic variation expressed in the 2 sexes (Clark and Begun 1998; Clark *et al*. 1999; Giardina *et al*. 2011). Ejaculate-female interactions may diverge quickly in *Drosophila* (Bono *et al*. 2011; Ahmed-Braimah *et al*. 2021; McCullough *et al*. 2022), likely under the influence of sexual selection. Even closely related allopatric populations of *D. mojavensis* show an exaggerated insemination reaction (Patterson and Stone 1952; Knowles and Markow 2001), which may contribute to reproductive isolation.

While the majority of the literature has, with good reason, focused on SFPs, seminal fluid contains other classes of molecules (reviewed in Hopkins *et al*. 2018). Three recent reports from dipterans, 1 from *Drosophila mojavensis* (Bono *et al*. 2011), 1 from *Drosophila novamexicana* (Ahmed-Braimah *et al*. 2021); and 1 from *Aedes aegypt*i (Alfonso-Parra *et al*. 2016), reported that RNA molecules are transferred from males to females during copulation. Whether this is a regulated or incidental phenomenon remains unknown, as does the possible biological significance of these RNAs (Leiblich *et al*. 2012; Corrigan *et al*. 2014). Some of these transferred RNAs appear to derive from accessory gland cells or exosomes (Leiblich *et al*. 2012; Corrigan *et al*. 2014). Here we use RNA-seq data in combination with natural genetic variation to investigate the prevalence and attributes of male-derived transcripts recovered from the seminal receptacle, spermatheca, and parovaria of mated *D. melanogaster* females. These organs are crucial to female reproduction, including ovulation, fertilization, and sperm storage. The parovaria, or female accessory glands, have secretory functions required for fertilization and ovulation (Sun and Spradling 2012); there is no evidence this organ stores sperm in *D. melanogaster*. The seminal receptacle and spermatheca do store sperm. The seminal receptacle is biased toward short-term sperm storage (Fowler 1973), and given the *D. melanogaster* mating system, may contain the sperm of multiple males (Manier *et al*. 2010). The paired spermatheca are biased toward long-term storage (Pitnick *et al*. 1999), and have secretory cells that participate in fertilization and ovulation (Schnakenberg *et al*. 2011; Sun and Spradling 2013).

## Materials and methods

### Data sources

The *D. melanogaster* inbred genotypes used here were strains from the Drosophila genetic reference panel (DGRP) collection (Mackay *et al*. 2012). The RNA-seq data from mated female reproductive tracts used in this study were from (Lombardo *et al*. 2023). In Lombardo *et al*. (2023), females from DGRP lines RAL 304, RAL 307, RAL 360, and RAL 399 were each crossed to males from RAL 517. Thus, males and females in each cross had different genotypes. Flies were raised at 25°C in a 12:12 light:dark cycle. For each female genotype, a single 3–5-day-old virgin female was placed in a vial with 2 RAL 517 males. On average 10 crosses were set up per pair of genotypes. Vials were observed, and following mating, males were removed—thus, in successful crosses the single female mated once. Three to five hours post copulation, when sperm storage organs are expected to be full (Fowler 1973), 3 female reproductive tract tissues were dissected; the parovaria, seminal receptacle, and spermatheca. All 3 female organs were obtained from RAL 304 and RAL 307, the seminal receptacle and spermathecae were obtained from RAL 360, and only the spermathecae were obtained from RAL 399.

Dissections were as described in Lombardo *et al*. (2023). Briefly, dissections were performed in 9-well depression plates; wells were filed with 1 ml ice-cold phosphate buffered saline (PBS). The order of dissection was as follows. The parovaria was removed from the rest of the reproductive tract and transferred to a new well. Connective tissue and fat were then removed before the parovaria were transferred to Trizol on ice. Next spermathecae were removed, rinsed in PBS, and transferred to Trizol on ice. Finally, the seminal receptacle was removed, rinsed in PBS, and then transferred to Trizol on ice. Forceps were checked for contamination between manipulating different organs and cleaned in ethanol.

RNA was extracted from dissected tissues using Trizol, and cDNAs were then produced using a SMART-Seq v4 Ultra Low Input RNA Kit for Sequencing (cat. 634896). Paired-end libraries were generated using the Nextera XT DNA Library Preparation Kit (FC-131-1024) and the Nextera XT Index Kit (FX-131-1001). Libraries were sequenced using 150 bp paired-end reads on an Illumina HiSeq4000 machine at the UC Davis Genome Center. The number of read-pairs generated per library and mean per base-pair coverage are in Supplementary Table 1. We obtained sperm transcriptome data from RNA-seq libraries reported in (Öst *et al*. 2014), which were downloaded from the Short Read Archive (PRJNA264746, downloaded 2023 June 3). This data set includes transcriptomes of mature sperm collected from seminal vesicles of flies raised on 2 different diets (2 replicates each). The dissected sperm were washed multiple times before RNA isolation. We aligned the RNA-seq reads from each sperm library to version 6.41 of the *D. melanogaster* reference (downloaded from Flybase.org 2021 August 9) using Hisat2 (Kim *et al*. 2015). StringTie (Pertea *et al*. 2015) was then used to estimate the abundance for each library.

### Identifying male-derived and female-derived molecules isolated from the female reproductive tract

Our approach used high-quality fixed single nucleotide polymorphism (SNP) differences between RAL 517 males and the female genotypes used in the experiment to identify transcripts that were isolated from the female, but which must have originated in the male. We downloaded the genotype file with SNP information for each genotype from (dgrp2.gnets.ncsu.edu/data.html, downloaded 2020 July 23). We updated the positions in this file from version 5 to version 6 of the *D. melanogaster* reference using (coords_r5_r6_major_arms.txt, downloaded from Flybase.org 2020 July 23, Gramates *et al.* 2022). We restricted our analysis to SNPs associated with only one gene (e.g. SNPs in overlapping exons on opposite strands were omitted) and removed regions of residual heterozygosity and regions masked in these genomes by Lack *et al*. (2015). We then identified exonic SNPs that distinguish RAL 517 from the female RAL genotype in each cross (Supplementary Table 2). Thus, a different set of potentially distinguishing sites (fixed SNP differences between genotypes) was identified for each cross. We then calculated the number of exonic SNPs per gene that distinguished RAL 517 from each female RAL strain to ascertain the maximum number of potential distinguishing SNPs that could be found for each gene and cross combination. We also calculated the mean sex-specific coverage of each SNP (Supplementary Table 2). We found that for crosses involving RAL 304 and RAL 307, male-specific coverage of distinguishing SNPs was higher than for crosses involving RAL 360 or RAL 399 (Kolgomorov–Smirnov tests *P* < 0.01 for all comparisons between crosses for the same tissue). However, because levels of overall coverage for each cross were similar (Supplementary Table 1), variation in male-specific coverage differences between crosses likely reflects a biological phenomenon rather than a technical artifact. For downstream analysis for each cross, we retained only genes that had at least 5 exonic SNPs that distinguished male and female genotypes and spanned ≥75% of the longest transcript's length (see below). This resulted in a different number of potentially distinguishable genes retained for analysis between each pair of genotypes.

To identify the parent-of-origin for transcripts recovered in the RNA-seq libraries, Bcftools (Danecek *et al*. 2021) was run to generate allele-specific coverage at each distinguishing site for each female reproductive tract RNA-seq library. For each library, we retained SNPs with a coverage ≥ 3 and required at least 3 calls for each allele at a site to infer the presence of reads containing each allele. We then took the longest transcript for each gene and inferred sex-specific coverage of that transcript by calculating the percentage of the transcript represented by the distance between the most proximal and distal distinguishing SNP identified as expressed in each sex. Given that sequencing of partially degraded transcripts, which may be over-represented in mature sperm (Ostermeier *et al*. 2002; Sendler *et al*. 2013) is expected to enrich for reads at the 3′ end in libraries created through polyA enrichment (as we have done here), we required 75% of a transcript to be spanned by sex-informative SNPs to reduce the potential contribution of degraded transcripts to our inferences. To reduce the probability of missing situations where variation in read alignment resulted in incorrect assumptions about transcript coverage, we performed a second round of transcript coverage calculations by aligning each library to a set of parent-specific mRNAs. To generate this mapping resource, we downloaded all mRNAs from FlyBase, kept the longest transcript for each gene, and then created a copy of this set for each genotype used in which we updated the genotype-specific file with SNPs identified in the DGRP-genotype file. Reads recovered from a mated female were aligned simultaneously to both genotypes involved in the cross.

The transcript coverage for genes identified as having produced a male- or female-derived transcript in at least 1 organ in a cross based on the distinguishing SNPs was updated for other organs of the same cross if the parent-specific reads indicated a minimum of 75% transcript coverage. The final list of genes associated with assignments of male- or female-derived transcripts was generated from the set of genes per library with ≥5 sex-distinguished SNPs at total coverage ≥15 summed over informative SNPs and exhibiting a minimum of 75% coverage of the longest transcript as defined by the locations of informative SNPs. These cutoffs were chosen to strike a balance between conservative calls and detecting transcripts that were not very abundant. Transcripts were then categorized as having been produced by female cells, male cells, or cells of both sexes.

To investigate enrichment of gene ontology terms we used functional annotation clustering in DAVID (Huang *et al*. 2009a, 2009b). The background gene list for the analysis consisted of genes that were detectable in 1 or more of our crosses using the detection criteria above.

## Results

Crosses between females of 4 different RAL genotypes and males from RAL 517 were dissected and the RNAs from up to 3 different female reproductive organs, spermatheca, parovaria, and seminal receptacle, were sequenced, with 1 library each per cross (Lombardo *et al*. 2023). An average of 7,477 genes per library were expressed at ≥1 transcript per million (TPM, a measure of relative abundance) (Supplementary Table 3). A total of 6,160 genes were associated with transcripts that were theoretically detectable as derived from the male or female in at least 1 cross (average 3,500 genes per cross), based on the number of distinguishing SNPs between genotypes and the inferred transcript length covered by the positions of the distinguishing SNPs (Supplementary Table 2); each of these 6,160 genes was theoretically detectable in an average of 2.3 of 4 crosses (dependent on the SNP differences between each pair of male and female genotypes).

**Fig. 1.**
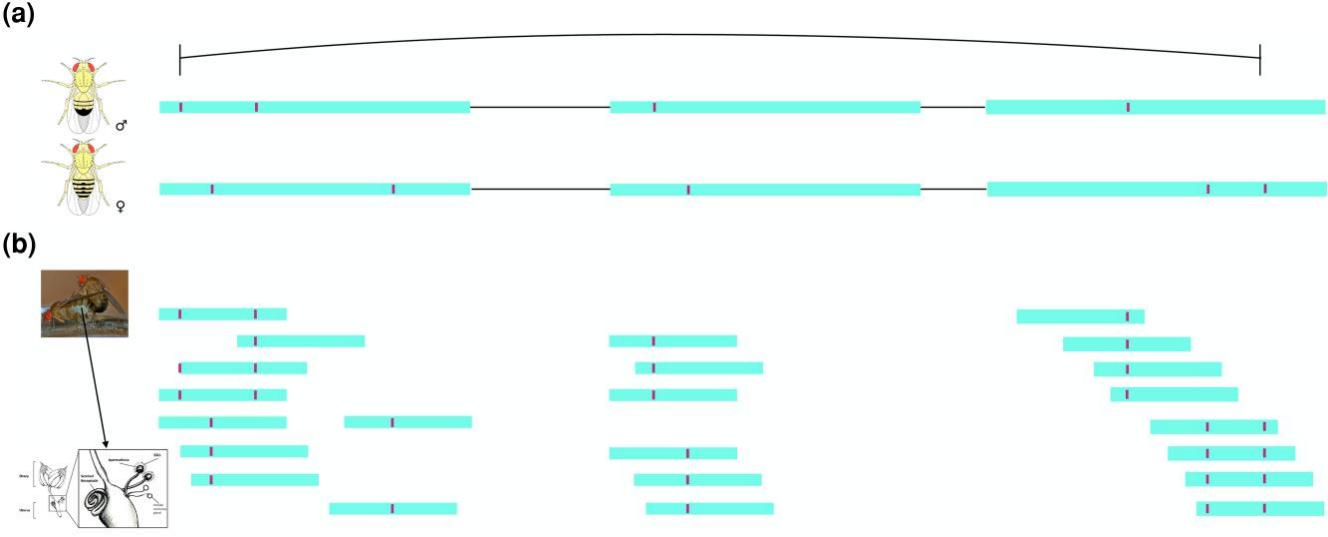
Identifying transcripts by parent-of-origin. a) Male and female genomes of each cross are examined to determine whether for the longest annotated transcript there are ≥5 fixed SNP differences between sexes that span at least 75% of the transcript. b) RNA-Seq reads from dissected reproductive tract organs of mated females are used to identify cases where there is coverage of ≥3x for ≥5 SNPs of ≥75% of the longest transcript. This is done for each organ and cross individually. The image in panel B of the female reproductive tract is from Wolfner (2011), female accessory gland = parovaria, SSCs, spermathecal secretory cells.

We then identified male- and female-derived transcripts in each RNA-seq library by imposing conservative coverage criteria—a minimum coverage of ≥15 counts (≥3 reads over ≥5 sex-informative SNPs) summed over distinguishing SNPs in the relevant libraries (Fig. 1. A set of 2,502 genes were detected using our criteria in 1 or more libraries, with the average gene meeting our filtering criteria in 4 of 9 libraries (Supplementary Table 4). This is a subset of the total genes expressed in these libraries. Only about 40% of genes expressed in our data are associated with transcripts exhibiting 5 or more sex-distinguishing SNPs that cover at least 75% of the length of the transcript and have a minimum coverage of 3 reads from those SNPs out of the total set of detectable genes (2,502/6,160) (Supplementary Table 4).

Of the genes that met our filtering criteria, we identified male-derived transcripts for 868 genes, with each male-derived transcript being identified in an average of 2.8 libraries (Table 1, Supplementary Table 5). Most genes associated with transcripts that were identified as male-derived, 81%, were also associated with evidence of female expression (Supplementary Table 4). Additionally, many genes that we categorized as producing male-derived, but not female-derived transcripts, were associated with weaker evidence of female-derived transcripts such that our criteria were not met (e.g. evidence of female-derived transcripts that did not cover sex-informative SNPs spanning 75% of the longest transcript). Thus, we view the identification of a male-derived transcript as a conservative positive assertion about the presence of a male transcript. Alternatively, we view evidence of absence of female-derived transcript to be more prone to false negatives, which seems sensible given that the primary focus in this work is identifying the constellation of male-derived molecules recovered from mated females. We investigated the expression of genes associated with male-derived transcripts in RNA-sequencing data from the testis and accessory gland + ejaculatory duct (AG) of RAL 517 (Cridland *et al*. 2020). Most genes corresponding to such transcripts (828/868, 95%) were expressed at a TPM of ≥1 in either RAL 517 AG or testis (Supplementary Table 5). None of the male-derived transcripts we recovered were from genes previously associated with such transcripts in *D. mojavensis* (Bono *et al*. 2011) or *D. novamexicana* (Ahmed-Braimah *et al*. 2021), though the number of putatively male-derived transcripts identified in those investigations was much smaller than the number identified here.

**Table 1.** Male-derived transcripts identified in reproductive tracts of RAL females mated to RAL 517 males.

Female genotype	Organ	Genes associated with male-derived transcripts
RAL 304	Parovaria	370
RAL 304	Seminal Receptacle	419
RAL 304	Spermatheca	299
RAL 307	Parovaria	425
RAL 307	Seminal Receptacle	447
RAL 307	Spermatheca	453
RAL 360	Seminal Receptacle	9
RAL 360	Spermatheca	7
RAL 399	Spermatheca	2
All crosses combined	All tissues combined	868

## Variation between organs and crosses

Substantial differences were identified between the crosses in terms of the number of genes for which male-derived transcripts were recovered. Many fewer genes were identified as having male-derived transcripts in crosses to RAL 360 and RAL 399 females; between 2 and 9 genes, compared to between 299 and 453 genes for libraries derived from RAL 304 and RAL 307 females. Given the lack of information regarding male-derived transcripts from RAL 360 and RAL 399 females, we will not consider them further.

We identified 108 genes associated with male-derived transcripts in 4 or more of the 6 libraries from RAL 304 or RAL 307. On average we observed male-derived transcripts from these genes in 5.4 out of 6 libraries (Supplementary Table 5) and for 64 genes, male-derived transcripts were observed in all 3 organs of RAL 304 and RAL 307 females (Supplementary Table 4). A male-derived transcript isolated from 4 or more libraries implies that it was recovered from both RAL 304 and RAL 307 females. These 108 genes are moderately abundantly expressed in reproductive organs of RAL 517 males (Supplementary Table 5); mean accessory gland + ejaculatory duct expression (TPM) = 17.7, and mean testis TPM = 19.6. About 60% of genes associated with male-derived transcripts in any organ within a cross, also had male-derived transcripts in all organs of that cross (Supplementary Table 6). Alternatively, only an average of 25% of genes with male-derived transcripts in an organ of either RAL 304 or RAL 307 females also had male-derived transcripts in female reproductive tract organs of the other genotype (Supplementary Table 7). Thus, it appears that male-derived transcript presence/absence variation is greater between crosses within an organ than across organs within a cross. Among the set of 64 genes associated with male-derived transcripts found in all 3 organs of both RAL 304 and RAL 307 were 11 identified as associated with the *D. melanogaster* sperm proteome (McCullough *et al*. 2022), 3 secondary cell markers of the accessory gland (Immarigeon *et al*. 2021), and 2 main cell markers of the gland (Majane *et al*. 2022) (Supplementary Table 5).

## Origin of male-derived transcripts

Given that the ejaculate is composed primarily of seminal fluid and sperm, it stands to reason that male-derived transcripts isolated from seminal receptacle, parovaria, and spermatheca, should primarily have their origin in the AG or sperm. AG-derived transcripts transferred to females in the seminal fluid may derive directly from secondary cells shed during mating (Leiblich *et al*. 2012 or from exosomes derived from secondary cells (Corrigan *et al*. 2014). To identify candidate male-derived transcripts recovered from RAL 304 and RAL 307 females that may have an AG source, we identified male-derived transcripts corresponding to SFPs (Wigby *et al*. 2020) or genes inferred to be biased toward secondary cell expression (relative to main cell; Immarigeon *et al*. 2021; Majane *et al*. 2022), main cell expression, or ejaculatory duct expression (Majane *et al*. 2022). Using these gene lists we found 50 genes potentially derived from the AG out of a total of 861 genes associated with male-derived transcripts recovered from RAL 304 and/or RAL 307 females (Supplementary Table 5). These genes include 12 SFPs (Wigby *et al*. 2020, 16 secondary cell markers (Immarigeon *et al*. 2021), 23 accessory gland markers, and 6 ejaculatory duct cell markers (Majane *et al*. 2022). The most consistently observed male-derived transcripts associated with possible AG function were 2 main cell markers from (Majane *et al*. 2022) *Prosap* and *CIC-a*, and 3 secondary cell markers (Immarigeon *et al*. 2021), *mrva*, *CG13887,* and *CG8460*, all of which were recovered from all 3 organs of RAL 304 and RAL 307 females.

The 50 genes associated with AG cell-biased expression were significantly enriched for secondary cell bias using both the (Immarigeon *et al*. 2021) and (Majane *et al*. 2022) genes lists (Table 2, Supplementary Table 5), though, only 3 genes, *mfas*, *nahoda,* and *Ttd14* (<20% of either list) overlap between the lists. In addition to the enrichment of secondary cell markers, we observed significant enrichment of ejaculatory duct cell markers from Majane *et al*. (2022). These roughly 2-fold cell-type enrichments are consistent with the idea that secondary cell transcripts are directly transferred to females, though the mechanism cannot be addressed using these data, and suggest the possibility that ejaculatory duct cell transcripts are also transferred to females.

**Table 2.** Enrichment of male-derived transcripts in gene lists^*a*^.

Origin of gene list	Type of gene list*^b^*	Male-derived transcripts in list	Total male-derived transcripts	Detectable genes in list	Total detectable genes	Male-derived transcripts in list/total male-derived transcripts	Detectable transcripts in list/total detectable transcripts	*P*(binom)
Wigby *et al*. (2020)	Seminal fluid proteins	12	866	89	4,847	1.39%	1.84%	0.803
Immarigeon *et al*. (2021)	AG Secondary cell markers	16	866	46	4,847	1.85%	0.95%	0.005
Majane *et al*. (2022)	AG Main cell markers	6	866	71	4,847	0.69%	1.46%	0.970
Majane *et al*. (2022)	AG secondary cell markers	17	866	50	4,847	1.96%	1.03%	0.005
Majane *et al*. (2022)	Ejaculatory duct cells	6	866	18	4,847	0.69%	0.37%	0.045
McCullough *et al*. (2022)	Sperm proteome	96	866	467	4,847	11.09%	9.63%	0.069

*^a^* Table includes only data from crosses to RAL 304 or RAL 307 females. *^b^*Some genes are present in multiple lists, see Supplementary Table 5 for details. AG, accessory gland.

To investigate more generally the possible contribution of sperm RNAs to the male-derived transcripts recovered from females we used an existing RNA-seq experiment carried out on mature sperm purified from seminal vesicles (Öst *et al*. 2014). Sperm were washed multiple times using insect media before RNA isolation. While this reduces the probability that incidental tissues (e.g. from the seminal vesicle) were carried through to the RNA extraction, it leaves open the possibility that some of the sperm-associated RNAs were derived from the outside of the sperm (e.g. via attached exosomes) rather than from the inside. We observed 6,908 genes with TPM ≥ 1 in all 4 mature sperm transcriptomes. For 703/868 (81%) of the genes corresponding to male-derived transcripts recovered from mated females, we also observed transcripts exceeding our abundance cutoff in all 4 mature sperm transcriptomes, suggesting that the majority of observed male-derived transcripts could originate from sperm (Supplementary Table 5). For the 703 genes, the mean abundance in the RAL 517 testis and AG transcriptomes was TPM = 35 and TPM = 26, respectively; we cannot distinguish the origin of these male-derived transcripts, sperm vs seminal fluid. Only 94 of these 703 genes were also identified as coding for components of the sperm proteome (McCullough *et al*. 2022). Thus, the proteins found in mature sperm often do not correspond to the transcripts found in mature sperm, consistent with the idea that the sperm proteins were largely synthesized earlier in development.

Given the read depth and transcript coverage criteria imposed on the RNA-seq data to identify candidate male-derived transcripts isolated from females, we sought to impose additional criteria on the sperm RNA-seq data that would facilitate a more straightforward comparison between the 703 genes and the sperm transcriptome data. We found that imposing a mean coverage per SNP of 2.25 (75% transcript length * 3 per nucleotide coverage) for the sperm transcriptomes, which is roughly similar to the criteria imposed on male-derived transcripts, corresponded to a minimum TPM of between 8.29 and 17.88 for each sperm RNA-seq library. This suggests that our criteria for detecting male-derived transcripts may be quite conservative and that the male-derived transcripts we recovered tend to be abundant in mature sperm.

Only 46/868 (5.3%) of male-derived transcripts fell below the abundance threshold in all 4 sperm RNA-seq libraries—these constitute stronger candidates for AG-specific origin (Supplementary Table 5). Of these 46 genes, 11 (24%) were expressed at a TPM ≥ 1 in the RAL 517 accessory gland + ejaculatory duct transcriptome. While many of these 11 genes appear to be fairly lowly expressed in the RAL 517 accessory gland, 3 were highly expressed. *CG30395* (TPM = 943), is a SFP (Wigby *et al*. 2020) and a main cell marker (Majane *et al*. 2022); male-derived transcripts of this gene were recovered from RAL 307 parovaria. Unfortunately, we have insufficient data to identify this transcript in other libraries. *Cyp4e3* was moderately abundant in the RAL 517 accessory gland (61 TPM) and male-derived transcripts were detected in both the parovaria and spermatheca of RAL 307 females. Male-derived transcripts were identified in all 3 organs of RAL 307 females for *Eglp2*, a secondary cell-biased gene identified by Immarigeon *et al*. (2021). This gene was moderately abundant in the RAL 517 AG transcriptome (19 TPM).

Of the 50 genes corresponding to male-derived transcripts that also were identified as exhibiting biased AG cell-type expression, 47 were also observed at TPM ≥ 1 in all 4 sperm RNA-seq libraries (see below). The correlation between AG cell-type biased transcripts and sperm transcripts make it challenging to determine the likelihood that transcripts of these 50 genes are of AG origin. The set of male-derived transcripts also observed in the sperm transcriptome includes 10 SFPs (Wigby *et al*. 2020), 15 secondary cell-biased genes (Immarigeon *et al*. 2021), and 27 cell-type markers as identified by Majane *et al*. (2022). One gene, *mfas*, is shared amongst all 4 of these lists and is very abundantly expressed in the AG (TPM = 1486). We find male-derived transcripts from this gene in the RAL 307 parovaria and find female-derived transcripts in all 3 organs for both the RAL 304 and RAL 307 females, as well as the seminal receptacle and spermatheca for RAL 360 females. The product of *mfas* is a cell adhesion protein—its possible function in the reproductive tract is unknown.

Notably, the parovaria do not store sperm in *D. melanogaster*. Nevertheless, male-derived transcripts from 18 genes found in the parovaria from 1 or both of RAL 304 and RAL 307 were also observed in all 4 sperm transcriptomes but expressed at a TPM < 1 in the RAL 517 AG (Supplementary Table 5). These genes include some relatively highly expressed in the RAL 517 testis including, *CG30431* (TPM 30.4), *ver* (TPM 25.2), and *Wnt2* (TPM 10.2). The parovaria were dissected first (Lombardo *et al*. 2023), before the seminal receptacle and spermatheca, making contamination of parovaria with sperm during dissection unlikely. Nevertheless, we investigated this possibility under the premise that sperm contamination would be most apparent for more abundant sperm transcripts. We observed that the sperm abundance of these 18 transcripts was no different from the average sperm transcript abundance (and in fact, were considerably less abundant), which does not support the contamination hypothesis. The presence of male-derived transcripts that were isolated from parovaria, yet appear to be strongly associated with sperm, is suggestive of the movement of male-derived products among the 3 focal organs. Our observation that there is more presence/absence variation of male-derived transcripts across crosses between organs than between organs within crosses is also consistent with this possibility.

## Genes with male- and female-derived transcripts

We observed 703 genes for which both female and male-derived transcripts were identified in the same library from RAL 304 and/or RAL 307 (Supplementary Table 4). Amongst these genes were 30 markers of AG cell types (Immarigeon *et al*. 2021; Majane *et al*. 2022), 78 components of the sperm proteome (McCullough *et al*. 2022), and 8 SFPs (Wigby *et al*. 2020).

Female-derived transcripts, either with or without a corresponding male transcript identified in the same library, were present in 1 or more libraries from RAL 304 and/or RAL 307 for 1,845 genes, though as noted earlier, there is evidence that many more genes are expressed in the female transcriptomes but did not satisfy our stringent criteria for assigning parental origin. Several genes (*n* = 70) were either identified as markers of AG cell types (Immarigeon *et al*. 2021) and/or (Majane *et al*. 2022) lists), code for components of the sperm proteome (174) (McCullough *et al*. 2022) or are SFPs (22) (Wigby *et al*. 2020) (Supplementary Table 4). Female-derived transcripts that code for components of the sperm proteome were observed in an average of 4/6 libraries; SFPs were observed in an average of 3.4/6 libraries and AG markers were observed in an average of 3.8/6 libraries. These findings support recent work suggesting that multiple proteins primarily thought of as functioning in male reproduction may also have a role in female reproductive functions (McDonough-Goldstein *et al*. 2021; McCullough *et al*. 2022).

## Abundant male-derived transcripts

We calculated the mean expression per male-derived transcript in each library by dividing the total coverage over all male-derived SNPs by the number of these SNPs to generate an estimate of coverage per male-derived SNP. Among the top 5% of such genes (*n* = 43 genes, Supplementary Table 8) the mean expression in lines where the gene is expressed was 152.6 reads/SNP, which is substantially higher than the average of 30.5 reads/SNP. These genes are categorized as present in an average of 3.2/6 libraries compared to 2.8/6 libraries for all genes. The gene with the highest expression, with a mean of 290 reads/SNP, is *Mdh2*. This gene product is part of the sperm proteome (McCullough *et al*. 2022) and is also highly expressed in the accessory gland of RAL 517, TPM = 156. There were 8 additional genes in this highly expressed set, the products of which are found in the sperm proteome. Two secondary cell markers (Immarigeon *et al*. 2021) and 1 SFP (Wigby *et al*. 2020, were also among this highly expressed set.

## Go analysis

We used DAVID to identify enriched functional annotation clusters amongst the male-derived transcripts relative to the set of detectable genes in our data set. We identified no enrichment amongst our 868 male-derived transcripts.

## Discussion

Identifying the intersexual molecular interactions related to internal fertilization is fundamental to investigations of the basic biology of sexual reproduction and to theories about the possible role of male–female interactions on polymorphism, divergence, and the evolution of reproductive isolation. Though it has been known for some time that male Drosophila may transfer RNAs to females during mating (Bono *et al.* 2011; Ahmed-Braimah *et al.* 2021), the identity of these molecules and their source are largely unknown. Here, using natural sequence variation, we have produced a catalog of male-derived molecules recovered from mated female reproductive tracts. Among the strongest results from this work is the finding of abundant sperm-associated RNAs that can be recovered from females. While several investigations have described mammalian sperm transcriptomes (Jodar *et al*. 2013), we were unable to identify *Drosophila* papers that used RNA-seq data to investigate the biology of transcripts carried by mature sperm. Given the presence of many degraded RNAs in mammalian sperm, which are thought to primarily be the detritus of spermatogenesis (Miller *et al*. 2005), we focused on detecting genes associated with male-transcripts that spanned most of the length of the longest annotated transcript. While such transcripts could simply reflect the tail of a gene-level transcript degradation distribution and so not necessarily be indicative of function, if there are functional sperm transcripts (Hosken and Hodgson 2014), our filtering should enrich them. The causal relationship, if any, between potentially sperm-derived RNAs recovered here and the sperm proteins recovered from mated females (McCullough *et al*. 2022) is an open question, though the apparent absence of cytoplasmic ribosomes in mature sperm (Sendler *et al*. 2013) suggests that the correlation is not likely functionally significant. More generally, the analyses presented here shed no light on whether or how the transcripts found in (or on) mature sperm might play a role in sperm function or early embryonic development. While relatively few paternal effect mutations have been identified in *D. melanogaster* (Fitch *et al*. 1997), the possibility remains that sperm RNAs have subtle effects that would often go undetected in most forward genetic screens.

Our result that cell-type markers of the AG are enriched among male-derived transcripts isolated from mated females is consistent with the notion that some male-derived transcripts in the female reproductive tract originate from secondary cells, perhaps via exosomes, as suggested by Leiblich *et al*. (2012) and Corrigan *et al*. (2014), or from ejaculatory duct cell exosomes, though we also recovered main cell markers. The observation that sperm-associated transcripts were recovered from the parovaria, an organ that does not store sperm, was unexpected. The fact that parovaria were dissected first would seem to disfavor the explanation that contaminating sperm from the dissection was the origin, though this cannot be ruled out completely, nor can the possibility that contaminating somatic tissues contributed to the sperm RNA isolated by Öst *et al*. (2014). However, another possible explanation for this observation is that molecules move between the focal organs studied here, the spermatheca, seminal receptacle, and parovaria. Parovaria-localized sperm-associated transcripts could plausibly originate in sperm-associated exosomes (Baskaran *et al*. 2020) that migrate to the parovaria, though this is highly speculative. More generally, however, the observation that many male-derived transcripts, including those originating from secondary cell marker genes, are found in both AG and sperm, means that a sperm origin for many male-derived transcripts cannot be ruled out with existing data. The correlations between AG and sperm transcriptomes (Cridland *et al*. 2020, 2022) mean that more incisive experiments, such as the direct transcriptome sequencing of AG-derived or sperm-associated exosomes, will be required to partition the possible origins and functions of male-derived transcripts isolated from mated females in Drosophila.

## Data Availability

Data was all acquired from publicly available resources. RNA-sequencing reads are from Lombardo *et al*. (2023) (PRJNA924827). Custom scripts used in the methods are available at https://github.com/JMCridland/FRT. Supplementary Tables are available through figshare: https://doi.org/10.25387/g3.24034332.
